# Influence of selected rootstock on growth parameters, accumulation of IAA and vitamins in scions of Cucumis sativus L. and Cucumis melo L.

**DOI:** 10.18699/vjgb-25-59

**Published:** 2025-07

**Authors:** A.Zh. Shoibekova, S.K. Jantassov, A.S. Jantassova, A.T. Samatov, T.S. Sagindykov, A.N. Karimova, G.A. Serikbayeva, M.R. Toishimanov, G.T. Bari

**Affiliations:** Kazakh National Agrarian Research University, Almaty, Kazakhstan; Kazakh National Agrarian Research University, Almaty, Kazakhstan; Kazakh National Agrarian Research University, Almaty, Kazakhstan; Kazakh National Agrarian Research University, Almaty, Kazakhstan; Kazakh National Agrarian Research University, Almaty, Kazakhstan; Kazakh National Agrarian Research University, Almaty, Kazakhstan; Kazakh National Agrarian Research University, Almaty, Kazakhstan; Kazakh National Agrarian Research University, Almaty, Kazakhstan Institute of Plant Biology and Biotechnology, Almaty, Kazakhstan; Kazakh National Agrarian Research University, Almaty, Kazakhstan

**Keywords:** morphometric analysis, grafting, C. maxima, HPLC, IAA, vitamins, морфометрический анализ, прививка, C. maxima, ВЭЖХ, ИУК, витамины

## Abstract

Grafting with resistant rootstocks is one of the most effective methods to prevent soil-borne diseases, and it can influence vegetative growth, flowering, maturation periods, and fruit quality, thereby ensuring high yields. In this study, four species from the family Cucurbitaceae were tested as potential candidates for grafting cucumber and melon: Cucurbita ficifolia Bouché, Cucurbita moschata L., Cucurbita pepo L. and Cucurbita maxima Duch. The study focused on the grafting methods that optimize growth parameters and the accumulation of hormones and vitamins in rootstock. The results indicated that Cucurbita maxima Duch. is the most suitable rootstock material for grafting to Cucumis sativus L. and Cucumis melo L., as it exhibited superior plant and root mass. Among the two grafting methods tested, the tongue approach (‘X’) demonstrated the best results in terms of growth parameters and the accumulation of indole-3-acetic acid (IAA) and vitamins in the scion leaves. IAA and vitamin concentrations were measured using HPLC in grafted samples at 2, 4 and 6 weeks of age. In the ‘X’ method, IAA accumulation from the end of the second week was twice as high compared to control plants. This method also showed higher vitamin content, with increased levels of B vitamins and vitamin C at the end of the 4th week (25.2–135.1 and 52.3–67.0 %, respectively), and vitamins A, E, D3 , K starting from the 2nd week (1.5–2 times higher). Conversely, the insertion or slant cut grafting method (‘Y’) did not show any significant increase in the analyzed parameters and was comparable to the control. The ‘X’ method for grafting both Cucumis sativus L. and Cucumis melo L. onto Cucurbita maxima Duch. plants demonstrated the best results and is recommended for production.

## Abstract (ru)

Прививка с помощью устойчивых подвоев – один из наиболее эффективных методов предотвращения болезней, передающихся через почву, который может влиять на вегетативный рост, цветение, периоды созревания и качество плодов, тем самым обеспечивая высокую урожайность. В настоящем исследовании четыре вида из семейства тыквенных были протестированы в качестве потенциальных кандидатов для прививки огурца и дыни: Cucurbita ficifolia Bouché, Cucurbita moschata L., Cucurbita pepo L. и Cucurbita maxima Duch. Исследование было сосредоточено на методах прививки, которые оптимизируют параметры роста и накопление гормонов и витаминов в привое. Согласно полученным результатам, отобранный вид Cucurbita maxima Duch. является наиболее подходящим материалом подвоя для прививки к Cucumis sativus L. и Cucumis melo L., поскольку он показал наилучшую массу растения и корней. Среди двух протестированных методов прививки язычковый подход (‘X’) продемонстрировал лучшие результаты с точки зрения параметров роста, накопления индолил-3-уксусной кислоты (ИУК) и витаминов в листьях привоя. Концентрации ИУК и витаминов измеряли с помощью ВЭЖХ в образцах привоя в возрасте 2, 4 и 6 недель. В методе ‘X’ накопление ИУК с конца второй недели было в два раза выше по сравнению с контрольными растениями. Этот метод также показал более высокое содержание витаминов, с повышенным уровнем витаминов группы В и витамина С в конце четвертой недели (25.2–135.1 и 52.3–67.0 % соответственно), а витаминов А, Е, D3, K – начиная со второй недели (в 1.5–2 раза выше). Напротив, метод прививки вставкой или косым срезом (‘Y’) не показал значительного увеличения анализируемых параметров и был сопоставим с контролем. Метод прививки ‘X’ как для Cucumis sativus L., так и для Cucumis melo L. на растения Cucurbita maxima Duch. продемонстрировал наилучшие результаты и рекомендуется для производства.

## Introduction

Grafting cucumber and melon (as scions) onto pumpkin rootstocks is one of the most effective methods to prevent soilborne diseases, influence vegetative growth, flowering, maturation periods, and fruit quality, thereby ensuring high yields of these crops (Mauro et al., 2022). Grafting vegetable crops is an important cultivation method in many countries where intensive and continuous cultivation is practiced (Farhadi et al., 2016), particularly in greenhouses with controlled conditions, where rootstocks can extend the fruiting period.

In global practice, rootstocks such as fig leaf gourd Cucurbita ficifolia Bouché (C. ficifolia) (El-Eslamboly, Deabes, 2014), winter squash landrace Cucurbita moschata L. (C. moschata) (Traka-Mavrona et al., 2000; Noor et al., 2019; Li X. et al., 2023), pumpkin Cucurbita maxima Duch. (C. maxima) (Farhadi et al., 2016), summer squash landrace Cucurbita pepo L. (C. pepo) (Noor et al., 2019), and combinations of C. maxima × C. moschata (Bekhradi et al., 2009; Toporek, Keinath, 2020) are used.

Scion-rootstock combinations affect pH, taste, sugar content, color, carotenoid content, fruit texture, resistance to low soil temperatures and salinity, and nutrient and water uptake. Studies have shown that RNA, proteins, and small molecules, some of which are involved in signal transduction, can move from the rootstock to the scion, directly affecting scion physiology (Mauro et al., 2022). This practice is also applied to other vegetable crops (Tsaballa et al., 2021). Such functional interdependence includes a complex relationship between the two plants, involving the exchange of water, nutrients, hormones, and other metabolites (Albacete et al., 2015).

Auxins play a central role in root formation. They induce the initiation of root primordia and influence the growth of newly formed roots. Plants produce indole-3-acetic acid (IAA) in shoot tips and young leaves, but exogenous auxin is important for successful rooting. There is no direct evidence that synthetic auxins can replace natural ones in cells, but they help in the overall accumulation of IAA in the plant, thereby promoting the formation of adventitious roots (Stefancic et al., 2007). The percentage of rooted cuttings positively correlates with the concentration of exogenous auxin, but only up to a certain point – at high concentrations, rooting stops or even decreases. Therefore, the presence of endogenous auxin in the plant is important (Stefancic et al., 2006).

The quality of the initial material plays a very important role in the formation of adventitious roots. The optimal physiological state of the initial plants can significantly improve the rooting of cuttings.

It is especially important to consider the accumulation of one of the main growth hormones, IAA (Balliu, Sallaku, 2017; Bunsangiam et al., 2021; Tang et al., 2023), in this case in scion-rootstock combinations (Noda et al., 2000; Li W. et al., 2017; Lam et al., 2020; Bantis et al., 2021) of cucumber and melon with pumpkins. In addition to resistance to biotic and abiotic factors, grafted plants need a good scion-rootstock union, rapid growth, and high productivity in a shorter time

Moreover, the accumulation of vitamins in plants as a biochemical indicator plays a significant role (Asensi-Fabado, Munné-Bosch, 2010; Abbas et al., 2023), particularly if these are water-soluble and fat-soluble vitamins with antioxidant properties (Asensi-Fabado, Munné-Bosch, 2010). Previously, the importance of their role in plants, various organs, and subcellular locations, as well as their main biosynthetic pathways, were described by the authors. In this context, it is necessary to study the influence of rootstock on scion in vitamin accumulation over post-grafting periods, as such studies have not been conducted previously according to the literature data. For optimal quantitative determination of IAA and vitamins, the high-performance liquid chromatography (HPLC) method is used (Battal, Tileklioğlu, 2001; Aslam at al., 2008; Keskin et al., 2022).

The aim of this study was to select the most suitable candidate from C. ficifolia, C. moschata, C. pepo, and C. maxima as rootstock for grafting of C. sativus and C. melo as scion, and to select the grafting method that ensures optimal growth parameters, measurement of IAA and vitamins in the scion.

## Materials and methods

Plant material. The plant material used for the study consisted of the following Cucurbitaceae species: cucumber (C. sativus) cultivar Asylim, melon (C. melo) cultivar Valet, fig leaf gourd (C. ficifolia) cultivar Arbuzny, winter squash landrace (C. moschata) cultivar Aphrodite, summer squash landrace (C. pepo) cultivar Danaya, and large-fruited pumpkin (C. maxima) cultivar Karina, from both Kazakhstan and global selections. The cultivation of cucurbits was conducted on neutralized peat with a pH of 6.0 (Kekkila™) in 1-liter containers with expanded clay drainage. Seeds of the cucurbits were planted in the peat-filled containers and watered daily with a nutrient solution of mineral salts at a rate of 100 mL per plant. After seed germination, the plants were illuminated with LED lamps at 5,000 lux for one week, followed by 10,000 lux for the subsequent six weeks. Morphometric analysis measures included plant mass and root mass separately, number and area of leaves, and stem thickness. Table 1 presents the composition of mineral salts and trace elements (According to the nutrient system of General Hydroponics, https://generalhydroponics.com).

**Table 1.**
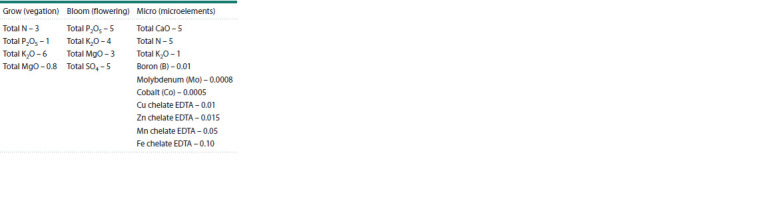
Minimum allowable composition of nutrient elements in stock solution, %

Every two weeks, the ppm values of the solution were increased by 500. To achieve concentrations of 500, 1,000, and 1,500 ppm in the nutrient solutions, 1.3 mL, 2.7 mL and 4 mL, respectively, were taken from each stock solution per liter (Table 1). The pH was adjusted to 6.0 using a 1M solution of NaOH or KOH. The total concentration of the nutrient solution was measured using a TDS meter.

HPLC analysis of IAA, fat- and water-soluble vitamins determination. The chromatographic separation was performed using a Shimadzu Prominence LC-20 system (Shimadzu, Japan) equipped with a UV detector (SPD-20A) and a fluorescent detector (RF-10AXL). The HPLC system was equipped with a binary pump (LC-20AD), an autosampler (SIL-20AC), a degasser (DGU-20A5) and a column oven (CTO-20A) controlled by LCSolution. For IAA, fat- and water- soluble vitamins determination were used the fresh plants.

Fat-soluble vitamins analysis. Stock solutions of vitamins A, D3, E, K 10 mg (Sigma Aldrich, USA) were dissolved in 10 mL of methanol in each falcon tube. Next, the working calibration standard was prepared with seven concentration ranges of 0.48–250 μg/mL for D3, 1.95–1,000 μg/mL for vitamin E, 0.195–100 μg/mL for vitamin K and 0.39–200 μg/ mL for vitamin A. All standards were stored at –20 °C and protected from light. The concentration of each vitamin was selected based on the sensitivity of the detector.

All working calibration standard solutions and samples were analyzed using the column Shimpack ODS XR (75 mm × 3.0 mm × 2.2 μm) (Shimadzu, Japan) and the following HPLC conditions: column oven temperature 35 °C; eluent Acetonitrile/ methanol/dichloromethane/H2O (70 : 15 : 10 : 5 %), and flow rate programmed using the following conditions: 0–10 min of 0.5 mL/min, then for 11–19 min the flow was increased to 1.0 mL/min, at 20 min it was decreased to 0.5 mL/min. Working calibration standards and samples were determined at a UV detector at 280 nm for 0–14 min, then the UV wavelength was switched to 295 nm. All these chromatographic parameters allow to better separate mixed standard calibrations.

In 1 g of the mixed sample, 100 μg ascorbic acid, 10 mL ethanol, 3 mL KOH (50 %) were added, stirred, and refluxed for 50 min using water bath at 80 °C. Extracts were neutralized with distilled water twice and then dehydrated using anhydrous sodium sulfate. Extracts were concentrated using rotary evaporator at 50 °C (IKA HB-8 basic, IKA, Germany), diluted by 5 mL acetonitrile, filtered (Aslam at al., 2008) using 0.45 μm membrane (Chromafil AO-45/25, Macherey-Nagel, Germany) and finally analyzed using HPLC.

Water-soluble vitamins analysis. The mobile phase consisted of 100 % acetonitrile and 99 % deionized water with 0.1 % orthophosphoric acid and 25 mM sodium dihydrogen phosphate. Flow rate was isocratic – 0.5 mL/min. The separation of vitamins was carried out in a Supelco Ascentis C18 column (250 mm – 4.6 mm – 5 μm) at 35 °C. Preparation of stock standard samples of B vitamins (B1, B2, B3, B5, B6, B7, B9, B12) was dissolved in deionized water at a concentration of 1 mg/mL. All water-soluble vitamins were purchased from Titan Biotech Ltd. All vitamins were pure and pharma-grade (purity at least ≥ 99 %). The solubility of vitamins B2 and B9 in water is limited, so a separate aqueous solution was prepared in 5 mM KOH and 20 mM KHCO3, respectively. Working standard samples will be prepared for B1, B5, B7 at a concentration range of 50–200 μg/mL, B3, B6 at 25–100 μg/ mL, B12 at 12.5–50 μg/mL, B2 and B9 at 2.5–10 μg/mL, and then all will be combined into one single standard for further calibration (Aslam et al., 2008).

For preparing an extraction solution, 50 mL of acetonitrile was mixed with 10 mL of acetic acid, and the final volume was made up to 1,000 mL with deionized water. 1 g samples were weighed and homogenized. After that, the samples were transferred into a conical flask where 10 mL of extraction solution was added. A water bath was set at 70 °C for 30 min. Afterwards, the sample was cooled down and finally filtered with filter trips (0.45 μm) and 20 μL aliquots solution was injected into the HPLC (Mozumder et al., 2019).

IAA analysis. Samples and standards were separated on a Restek Ultra C18 HPLC column 150 mm × 4 mm, 5 μm (Bellefonte, PA, USA) at 40 °C. UV detection was performed at 269 nm. The flow rate of the mobile phase was 0.8 mL/min. Mobile phase A consisted of 100 % HPLC grade acetonitrile, mobile phase B consisted of 99.9 % HPLC grade water and 0.1 % formic acid using the gradient elution as follows: 95 % B, 0 min; 70 % B, 13 min; 95 % B, 15 min. The flow rate of the mobile phase was 0.8 mL/min (Battal, Tileklioğlu, 2001; Keskin et al., 2022). 10 mg of IAA standard solution was dissolved in 1 mL 1N NaOH, then filled with 9 ml deionized water in a 10 mL tube.

1 g samples were weighed and homogenized. After that, the samples were transferred into 10-mL centrifuge tubes and 10 mL of acetonitrile was added: deionized water (9 : 1, v/v) under dim light conditions. Then, 100 μL formic acid was added and shaken. Homogenates were incubated for 2 h, and centrifuged at 12,000g at 4 °C for 20 min. The upper supernatants were then collected and dried in a vacuum evaporator (Biobase, China). The residues obtained by drying were dissolved in 2 mL of acetonitrile and purified by a 200 mg/3 mL C18 solid phase extraction cartridge (Strata, Phenomenex, Torrance, USA). The cartridge was prepared by successively passing 2 mL of water and 2 mL of ethanol using SPE tube vacuum manifold (Biobase, China), where the vacuum valve was set at negative pressure – 0.01 MPa. The liquid that passed through the cartridge was discarded, and the IAA was washed off with 2 mL of a mixture of ethanol–water–formic acid (80 : 20 : 0.5 %; v/v) into a 10 mL centrifugal tube. The collected residuals were transferred to a 2 mL Eppendorf tube, evaporated using a sample concentrator (NDK200-2N, Miulab, China), then dissolved with 2 mL acetonitrile. Finally, 20 μL aliquots solution was injected to HPLC.

Grafting methods. Two of the most common grafting methods were used for plant grafting. The first method, termed ‘Y’, involved insertion grafting or slant-cut grafting. One cotyledon leaf was left on the rootstock to enhance grafting success (approximately to 90 %). All leaves, including cotyledon leaves, were left on the scion. The graft junction was secured with a clip.

The second method, termed ‘X’, involved tongue approach grafting. Longitudinal cuts at an angle of 20–30° were made on the stems of both the rootstock and the scion, with the cut on the rootstock directed downward and the cut on the scion directed upward. The plants were then joined by their tongues and secured with clips. Initially, both root systems were used. After 10 days, the vegetative part of the rootstock and the root system of the scion were pruned.

The grafted plants were then placed in a climate chamber with controlled temperature, humidity, and light conditions for further grafting. The grafted plants were grown in the growth chamber at 20 °C with 92 % humidity in complete darkness for four days. Subsequently, the grafted plants were grown under 12,000 lux lighting with an 8/16-hours light/dark cycle for 10 days (Lee et al., 2010). C. sativus and C. melo were used as control and self-grafted. All data were statistically assessed using Duncan’s test.

## Results

**Morphometric analysis of the Cucurbitaceae family**

**Fig. 1.**
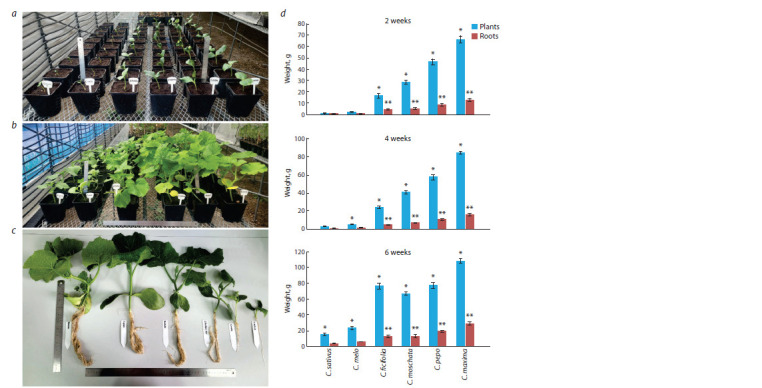
Growth of Cucurbitaceae plants: a, one-week seedlings after germination: b, four-week plants; c, morphometric analysis of the Cucurbitaceae plants; d, 2-, 4- and 6-week plants and roots’ weight in gram of fresh plants. * Statistically significant results between the Cucurbitaceae plants at p ≤ 0.05. ** Statistically significant results between the Cucurbitaceae roots at p ≤ 0.05.

For setting up the experiment on growth parameters (plant and root mass), seeds of rootstocks C. maxima, C. pepo, C. ficifolia, and C. moschata, and scions C. sativus and C. melo were sown for further morphometric analysis and extraction (for quantitative determination of auxins and vitamins) over a period of 2 to 6 weeks (Fig. 1а). For reliable results, experiments were conducted in three replicates under identical conditions: seedlings were planted in 1,000 mL containers and irrigated with a nutrient solution at a concentration of 500 ppm. During the third and fourth weeks, the concentration of the nutrient solution was increased to 1,000 ppm, and from the fifth week to the end of the sixth week, it was increased to 1,500 ppm. The results showed a significant difference across the four types of rootstocks and two samples of scions.

As an example, Figure 1b shows the plants at four weeks post-germination and the morphometric analysis (Fig. 1c). The morphometric analysis focused on the mass of the entire plant and the root. The results of the morphometric analysis are presented in Figure 1d as bar charts

At 2, 4, and 6 weeks post-germination, plant and root mass parameters were measured using digital scales adapted from the methods. Based on the plant and root mass measurements at all intervals (2, 4, and 6 weeks), C. maxima exhibited the highest values. C. pepo ranked second in these metrics. However, at the 6-week mark, C. ficifolia matched C. pepo in plant mass, though all its values remained significantly lower than those of C. maxima. The lowest values were observed in C. sativus and C. melo. Morphometric analysis identified C. maxima as the most suitable candidate for grafting of C. sativus and C. melo. C. sativus plant did not show statistical significance, except for the 6th week. C. melo, C. ficifolia, C. moschata, C. pepo, and C. maxima plants showed significant differences, with C. maxima having the highest plant biomass over time. C. sativus and C. melo roots weight had no statistical significance at all. C. ficifolia, C. moschata, C. pepo, and C. maxima root weights showed significant differences.

**IAA, water- and fat-soluble vitamins in grafted plants**

**Fig. 2.**
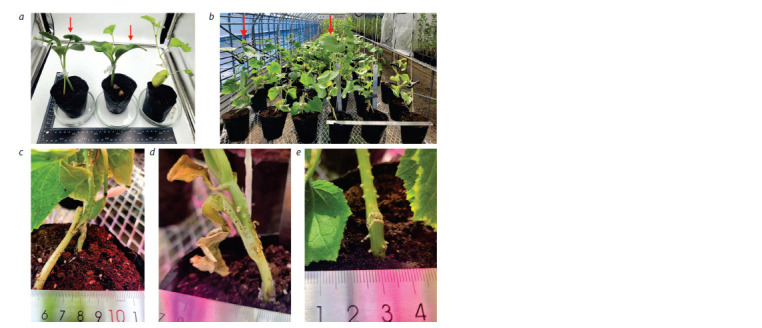
Grafting of C. sativus and C. melo on C. maxima. a, From left to right X-type grafting, Y-type grafting and self-grafting accordingly; b, grafted and self-grafted Control plants; c, X-type grafted connection between scion and rootstock; d, Y-type grafted connection between scion and rootstock; e, self-grafted Control.

In the second stage, grafting of one-week-old plants of C. sativus and C. melo onto the pumpkin rootstock C. maxima was performed. The grafting method is illustrated in Figure 2a. The tongue approach grafting (Fig. 2a, first from the left) is designated as ‘X’ based on the shape of stem fusion. The second grafting method (second from the left) involved hole insertion grafting (scion stem with an oblique cut) to the growth point of the rootstock and is conditionally designated as ‘Y’. For the ‘Y’ grafting method, the true leaf buds of the rootstock were removed, and a cut was made along the stem where the scion with an oblique stem cut was attached. The grafting methods ‘X’ and ‘Y’ are marked with red arrows in Figure 2a. The third grafting method was the control, where a tongue cut was made on the C. sativus and C. melo plants to ensure the experiment was conducted under the same conditions. The self-grafted scion plants of C. sativus and C. melo are designated as Control.

**Fig. 3.**
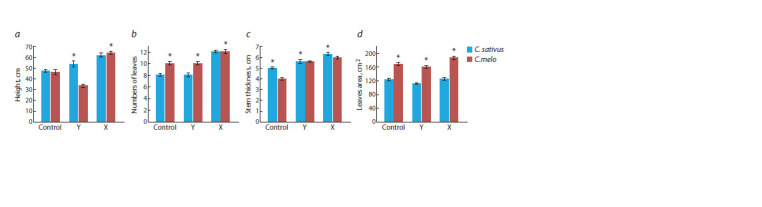
Morphometric analysis of 6-week-old grafted plants (fresh plants): a, plant height; b, number of every grafted plant leaf; c, stem thickness; d, leaves area of grafted plants. * Statistically significant results between the control and experimental groups at p ≤ 0.05.

Two weeks after grafting, active plant growth commenced. Differences in growth rate and development between the grafted plant variants were observed at the end of the third week. Figure 2b illustrates the results for plants at four weeks of age marked with red arrows. Additionally, the healed graft unions are shown in Figure 2c–e for graft variants ‘X’, ‘Y’, and Control, respectively. From the start of grafting until the end of the sixth week post-grafting, it was visually observed that the graft variant ‘X’ did not lag behind the control plants and the ‘Y’ variant. In the graft variant ‘Y’, a slowdown in growth and development was noted. Upon reaching six weeks of age, a structural analysis was conducted, which identified the graft variant ‘X’ as the best among the others (Fig. 3). Six weeks post-grafting, the ‘X’ variant exhibited a 96 % survival rate, while the ‘Y’ variant showed only a 43 % survival rate of grafted plants. The control variant also demonstrated a high survival rate of 97 %.

**Fig. 4.**
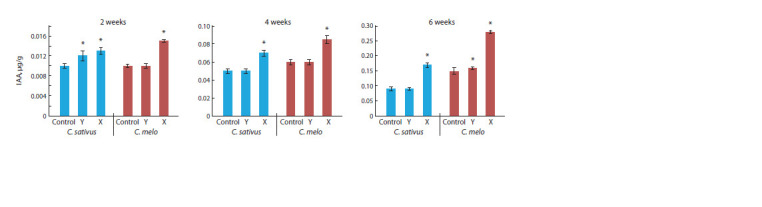
IAA content in C. sativus and C. melo depending on grafting methods (from fresh plants). * Statistically significant results between the control and experimental groups at p ≤ 0.05.


Upon reaching six weeks of age, a structural analysis of the grafted plants was conducted, revealing that the grafting variant ‘X’ was superior to the others (Fig. 3). This variant excelled in plant height, leaf number, stem thickness, and leaf area for both C. sativus and C. melo. Grafting variant ‘Y’ ranked second in stem thickness and matched the Control in leaf number and leaf area. When comparing C. sativus and C. melo, no significant differences were found within the ‘X’ variant of grafting, for plant length, stem thickness and number of leaves, except for leaves area according to Figure 3. The IAA content in the grafted plant variants is presented in Figure 4. The extracts of leaves and stems were filtered through solid phase extraction (SPE) (Supplementary Fig. S1)1. Upon completion of the extraction, IАА detection was performed, which was expressed as a chromatographic peak (Fig. S1). Also, chromatographic peaks of water and fatsoluble vitamins quantification of the Cucurbitaceae family are shown in Supplementary Figure S2. And peaks of pure standard substances of IAA, water/fat-soluble vitamins are provided in Supplementary Figure S3

Supplementary Materials are available in the online version of the paper: https://vavilovj-icg.ru/download/pict-2025-29/appx20.pdf


Based on the results of the HPLC analysis, it was found that the content of IAA and vitamins is higher in the leaves than in the stems. Consequently, we conducted further targeted biochemical analyses using the leaves. As a result, the highest accumulation of IAA was observed in the graft variant ‘X’. As shown in Figure 4, the accumulation of IAA in grafted plants increased in the ‘X’ variant from the end of the second week, and by the end of the sixth week, the difference with the control plants was almost doubled.

Vitamins identification using chromatographic detection was performed (Tables 2, 3). The quantitative values of vitamins are given in μg/g, recalculated to dry weights of stem and leaf samples. The differences in the content of water- and fat-soluble vitamins in the grafted plant variants are presented in Tables 2, 3. A significant increase in vitamin content was observed from the end of the fourth week.

**Table 2.**
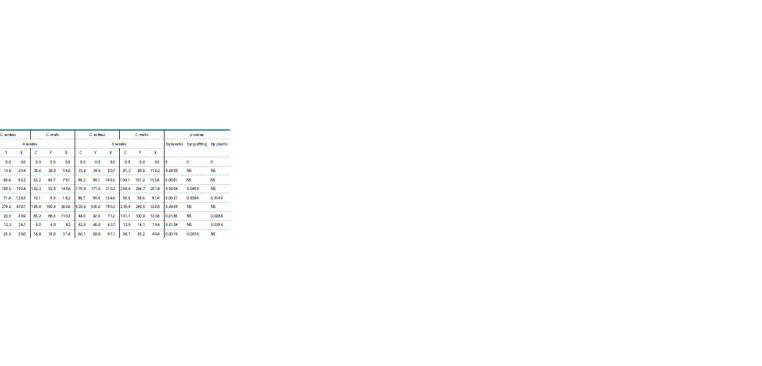
Water-soluble vitamins accumulation in grafted plants (from fresh plants), μg/g Note. Here and in the Table 3: C – control, Y – ‘Y’ type of grafting, X – ‘X’ type of grafting.

**Table 3.**
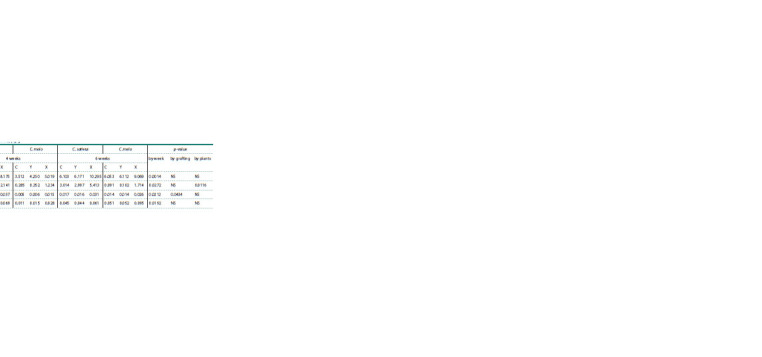
Fat-soluble vitamins accumulation in grafted plants (from fresh plants), μg/g

An increased content of all B vitamins and vitamin C was noted in the grafting variant ‘X’ at the end of the 4th week – 25.2–135.1 and 52.3–67.0 %, respectively, with all indicators being higher in the grafted C. sativus than in the C. melo. In C. melo, vitamin B2 levels exceeded the Control by 122.7 %, and those of vitamin B12, by 135.1 %. In the grafting variant ‘Y’, all indicators were at the control level.

After 6 weeks, the indicators in the grafting variant ‘X’ became more balanced and amounted to 17.5–61.8 % for B vi70 tamins and 51.6 % for vitamin C in C. sativus; 19.3–42.6 % for B vitamins and 44.9 % for vitamin C in C. melo. In the grafting variant ‘Y’, the indicators were also at the standard Control level.

For vitamins A, E, D3, and K, starting from the 2nd week, a stable difference in their accumulation in leaves was observed in the grafting variant ‘X’, being 1.5–2 times higher compared to the Control variant.

Additionally, in the ‘X’ variant, the accumulation of IAA compared to the control plants in Control also increased from the end of the second week and was almost twice as high. This pattern with the grafting variant ‘X’ was observed both in the C. sativus grafting variant and in the melon grafting variant.

## Discussion

The influence of the root system of the rootstock on the scion, and vice versa, i. e., the impact of the more developed biomass of the rootstock on the further development of the scion and improved growth of melon plants grafted onto Cucurbitaceae species, was identified in a previous study (Martínez-Ballesta et al., 2010). This study confirms that the union of the rootstock and scion and the differentiation of new vascular tissue from callus cells, as well as the resumption of scion biomass growth, begin within 2 weeks post-grafting, as clearly demonstrated in our research.

Based on the obtained results, we observe that the grafting method ‘X’ is the most acceptable among other methods. As expected, this method allows plants to resume growth processes more quickly and better accumulate IAA and vitamins in the leaves. According to (Noor et al., 2019), the use of the tongue grafting method showed high compatibility with hybrid cucumber scions compared to other grafting methods and non-grafted plants.

The novel idea of the study was to identify the optimal type of grafting that will result in the fastest recovery of the grafted plant, as well as a stimulating effect of the rootstock on the scion, where an increase in IAA and vitamin content occurs. Other researchers have noted that the influence of cucurbit rootstock on cucumber scion provides salt tolerance and increases fruit yield by improving morpho-physio-biochemical and ionic properties, specifically increasing the content of the following substances in grafted plants: superoxide dismutase, catalase and peroxidase enzymes, antioxidant scavenging activity, ionic ↑ K and Ca, ↓ Na (Abbas et al., 2023). We obtained similar results. It can be assumed that the mechanism of vitamin accumulation in scions occurs similarly to the mechanism of IAA accumulation in scions post-grafting onto the rootstock. In other words, the growth factors of the rootstock in the form of IAA may stimulate the accumulation of hormones and vitamins in the scion. The conducted studies, including morphometric analysis of grafted plants, show that parameters such as plant height, number of leaves on grafted plants, stem thickness, leaf area of grafted plants, as well as chromatographic data on IAA and vitamin accumulation, are superior in grafting method ‘X’ compared to the control and grafting method ‘Y’.

The next stage should include studies throughout the entire physiological development of the plant, up to full maturation and harvest.

## Conclusion

Analyzing the data, we concluded that among four species of Cucurbitaceae: C. ficifolia, C. moschata, C. pepo and C. maxima, the plants of the species C. maxima are the best candidate for use as rootstock material for grafting of C. sativus and C. melo. This is due to their superior performance in terms of plant mass increase, root mass, and stem thickness at the root base for both C. sativus and C. melo. The second-best candidates are plants of the species C. pepo.

Among the two different grafting methods tested, the grafting method ‘X’ showed the best results in terms of growth factors and the accumulation of IAA and vitamins in the leaves of the rootstock. In method ‘X’, the IAA accumulation from the end of the second week was twice as high compared to the Control plants. Regarding vitamins, this method also exceeded the control, with increased levels of all B vitamins and vitamin C at the end of the fourth week by 25.2–135.1 and 52.3–67.0 %, respectively, and vitamins A, E, D3, and K starting from the second week by 1.5–2 times. In contrast, the grafting method ‘Y’ did not show any significant increase in any of the analyzed parameters and was at the Control level.

Therefore, it is recommended to graft both C. sativus and C. melo onto C. maxima plants using the tongue approach grafting method ‘X’.

## Conflict of interest

The authors declare no conflict of interest.
